# Distributed structure determination at the JCSG

**DOI:** 10.1107/S0907444910039934

**Published:** 2011-03-18

**Authors:** Henry van den Bedem, Guenter Wolf, Qingping Xu, Ashley M. Deacon

**Affiliations:** a Joint Center for Structural Genomics, Stanford Synchrotron Radiation Lightsource, SLAC National Accelerator Laboratory, Menlo Park, CA 94025, USA

**Keywords:** distributed protein-structure determination, consensus models, parallel computing

## Abstract

The Joint Center for Structural Genomics (JCSG), one of four large-scale structure-determination centers funded by the US Protein Structure Initiative (PSI) through the National Institute for General Medical Sciences, has been operating an automated distributed structure-solution pipeline, *Xsolve*, for well over half a decade. During PSI-2, *Xsolve* solved, traced and partially refined 90% of the JCSG’s nearly 770 MAD/SAD structures at an average resolution of about 2 Å without human intervention. *Xsolve* executes many well established publicly available crystallography software programs in parallel on a commodity Linux cluster, resulting in multiple traces for any given target. Additional software programs have been developed and integrated into *Xsolve* to further minimize human effort in structure refinement. *ConsensusModeler* exploits complementarities in traces from *Xsolve* to compute a single optimal model for manual refinement. *Xpleo* is a powerful robotics-inspired algorithm to build missing fragments and *qFit* automatically identifies and fits alternate conformations.

## Abstract 2

The software suite *Xsolve* semi-exhaustively explores key parameters of the X-ray structure-determination process to compute multiple three-dimensional protein structures independently and in parallel from a set of diffraction images. An optimal consensus model for subsequent manual refinement is computed from these structures.

## 1. Introduction   

The Protein Structure Initiative is a national effort in the USA to determine a large collection of three-dimensional protein structures in a high-throughput operation, with the long-term objective of making the atomic level details of most proteins easily obtainable from their corresponding DNA sequences. In the pilot phase, which ran from 2000 to 2005, new approaches and tools were developed to streamline and automate the steps of protein structure determination. In the subsequent production phase, which ended in mid-2010, these high-throughput methods resulted in a large number of unique protein structures. Since its inception in 2000, the Joint Center for Structural Genomics (JCSG) has focused on the development of methodologies and protocols to automate and streamline its structural genomics process, from target selection and protein expression to ultimately the deposition of high-quality three-dimensional structures in the Protein Data Bank (PDB; Berman *et al.*, 2003 ▶).

Here, we present the software suite *Xsolve*, which was developed by the JCSG to automatically execute all steps of the X-ray structure-determination process from reading diffraction images to calculating a partially refined three-dimensional model and thus reduce the need for human intervention. Fully automating routine tasks associated with solving a structure allows the JCSG to focus its efforts on more demanding aspects of structure determination, ensuring that a high-quality structure will be produced. In contrast to pre­viously reported automated structure-determination methods (Holton & Alber, 2004 ▶; Fu *et al.*, 2005 ▶; Panjikar *et al.*, 2005 ▶; Adams *et al.*, 2010 ▶), *Xsolve* was designed to explore key parameters of protein structure determination independently and in parallel across all stages of the process. It also employs multiple software programs for identical tasks at each stage. Many ‘promising’ parameter and program combinations are simultaneously carried forward to the final stages, resulting in many structures with varying degrees of completeness and accuracy. While many processing strategies lead to good protein structures, there are numerous cases in which some strategies fail or are substantially outperformed by others. All final structures are then collected by *ConsensusModeler*, which exploits their complementarities by computing a consensus model that serves as an optimal starting point for subsequent manual refinement.

## 2. Methods   

*Xsolve*’s design reflects a distributed data-driven approach to solving protein structures; rather than trying to execute a single best strategy to arrive at an optimal set of initial co­ordinates (a ‘trace’) for further refinement, it explores many independent trials in parallel. *Xsolve* computes a ‘tree’ of solutions, with the traces (the leaves of the tree) being the end result of a long sequence of branch points throughout the structure-determination process. The distributed tasks are then reduced into a final trace. This general architecture, which is commonly used in modern computing, is highly robust to failure from suboptimal processing or hardware malfunction.

### 2.1. *Xsolve*    

**Figure 1:**
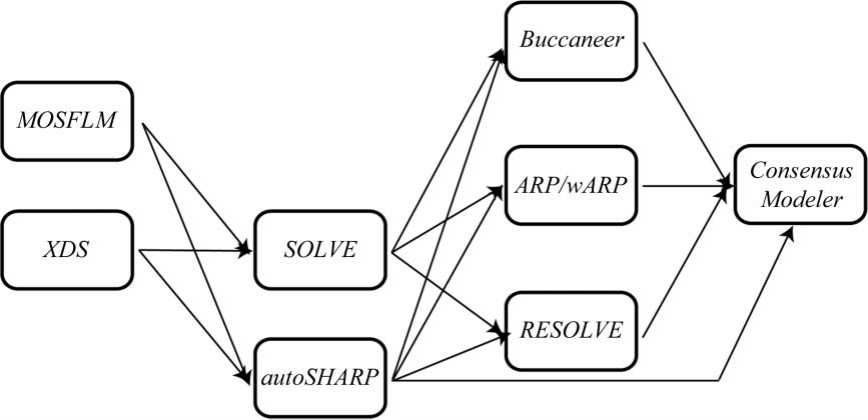
Parallelization at the ‘program level’ in *Xsolve*. All outputs at each stage of *Xsolve* are distributed independently and in parallel to all programs at the next stage. Shown here are 14 combinations of software programs at the three stages in structure determination. *autoSHARP* includes model building with *ARP*/*wARP* and the resulting models are collected by *ConsensusModeler*. *autoSHARP* phases are input to *Buccaneer* and *RESOLVE*.

*Xsolve* implements all processing steps required to compute an electron-density map and a trace from diffraction images. It executes well established third-party software programs for data reduction (indexing, integration and scaling), phasing and tracing of the experimental maps in succession without human intervention. The third-party software programs currently included are *MOSFLM* (Leslie, 1992 ▶), *XDS* (Kabsch, 2010 ▶) and *HKL*-2000 (Otwinowski & Minor, 1997 ▶; for testing only) for data reduction; *SHELXD* (Sheldrick, 2008 ▶), *SOLVE* (Terwilliger & Berendzen, 1999 ▶) and *autoSHARP* (Vonrhein *et al.*, 2007 ▶) for heavy-atom location and phasing; and *ARP*/*wARP* (Perrakis *et al.*, 1999 ▶), *RESOLVE* (Terwilliger, 2003 ▶) and *Buccaneer* (Cowtan, 2006 ▶) for building a model into the electron-density map. Fig. 1 ▶ depicts the flow of information in *Xsolve*.

*Xsolve* can also solve a data set using molecular replacement (MR). A protocol similar to that reported in Schwarzenbacher *et al.* (2008 ▶) was implemented. Parameters such as multiple MR templates, resolution cutoff and space groups are explored in parallel using multiple MR programs: *MOLREP* (Vagin & Teplyakov, 2010 ▶), *EPMR* (Kissinger *et al.*, 1999 ▶) and *Phaser* (Storoni *et al.*, 2004 ▶). Potential MR solutions are subjected to rigid-body refinement and restrained refinement with *REFMAC* (Murshudov *et al.*, 1997 ▶). Model rebuilding is carried out using *ARP*/*wARP* and *RESOLVE*. As the JCSG uses single-wavelength or multiwavelength anomalous diffraction (SAD/MAD) techniques to obtain phases for the vast majority of its targets, the remainder of this paper will focus on SAD/MAD data.

Once a data set has been collected, a crystallographer completes a simple web form to inform the system of a few parameters, such as the location of the diffraction images, the resolution limit and theoretical or experimentally determined anomalous scattering factors. Optionally, to limit the search space, space groups and the number of monomers in the asymmetric unit can be provided as input to *Xsolve*. The data-collection strategy is reconstructed from parsing diffraction-image headers. Additional parameters, such as the amino-acid sequence, molecular weight and heavy-atom information, are automatically read from a database. The molecular weight can also trivially be derived from the sequence. Once this is complete, the job can be submitted to *Xsolve* with a different web form. The status of *Xsolve* can also be checked within a browser. Screenshots of the three web forms are included as supplementary material^1^ to this paper.

#### 2.1.1. Parallelization   

Independent trials are executed in parallel on a 300-core compute cluster. Parallelism is employed at the ‘data level’ and at the ‘program level’. At the ‘data level’ multiple space groups, (MAD) wavelength combinations and number of monomers per asymmetric unit are sampled. For instance, a crystallographer can instruct *Xsolve* to solve the structure in space groups *P*2_1_ and *P*2_1_2_1_2_1_ to account for possible higher metric symmetry. Wildcards are also accepted, so that *P*2* will be expanded by *Xsolve*. Unless explicitly overridden, *Xsolve* will attempt to solve the structure by sampling a number of monomers per asymmetric unit compatible with the estimated solvent content of the unit cell. The JCSG typically collects MAD data at three wavelengths whenever possible. All wavelength combinations are explored simultaneously from the data-reduction step on to evaluate the best phases independent of possible radiation damage to the crystal. Similarly, at the ‘program level’ all combinations of the third-party software programs are explored in parallel. For instance, whenever a wavelength/space-group combination is output at the integration stage, it serves as input to all programs at the phasing stage. When one of the phasing applications outputs an experimental map corresponding to a wavelength/space group/monomer combination it serves as input to all model-building programs. Fig. 1 ▶ displays 14 com­binations of software programs at the three major stages of structure determination. Assuming a fixed choice of space group and a fixed number of monomers per asymmetric unit, together with six wavelength combinations, this already leads to 72 processing strategies. In practice, many strategies are easily determined to be suboptimal and are pruned at an early stage. The resulting traces are ultimately collected by *ConsensusModeler* and condensed into a single optimal trace.

#### 2.1.2. Implementation   

*Xsolve* was implemented in Java following a master/worker model. Each compute core of the cluster is associated with a worker module that communicates with a central ‘master’ server module. The server generates jobs, which it holds in a queue for the worker nodes to process. The third-party software programs are started from shell scripts that are generated dynamically at each stage. These shell scripts are generally very simple, consisting of a call of the program for the next processing step together with the input parameters that were determined in the preceding processing steps. Information flows from one program to the next by means of Extensible Markup Language (XML) intermediary format files (W3C World Wide Web Consortium; http://www.w3.org/XML/). Upon the successful completion of a worker task, the Java execution environment parses the task log file and stores the values of pertinent parameters in XML format. The correct parameters are then imported into the next task’s shell script. This XML-driven architecture facilitates manual intervention at any stage, if desired, and in addition completely decouples job scheduling and execution from the crystallographic workflow; new processing stages are easily added by modifying a shell script template.

### 2.2. *ConsensusModeler*: combining traces   

**Figure 2:**
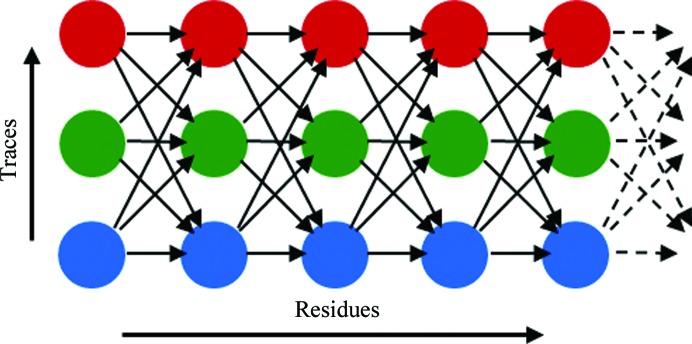
Superimposed traces represented as a graph. A docked trace is represented from left to right; traces resulting from different model-building protocols are represented on the vertical axis.

*ConsensusModeler* capitalizes on *Xsolve*’s exploration of model building by combining traces output by different strategies to obtain a more complete and error-corrected trace. *ConsensusModeler* collects *Xsolve* traces that have more than 40% of side chains docked into the density. Trace errors for models not meeting this threshold tend to be severe. The *ConsensusModeler* algorithm first superimposes traces using crystallographic symmetry operators, automatically shifting the origin or re-indexing the data wherever necessary. Next, NCS-related traces are superimposed using *SSM* (Krissinel & Henrick, 2004 ▶). The *ConsensusModeler* algorithm accepts the sequence (side-chain identity) assignment from each of its input traces; any conflicts will be, by its nature, optimally resolved by the algorithm. Undocked fragments are set aside. Each superimposed trace is represented in a graph, with each residue corresponding to a vertex (Fig. 2 ▶). Vertices (residues) from all contributing input traces are connected by directed edges such that a residue with sequence number *i* − 1 from any subunit in any trace *j* is connected to all residues with sequence number *i* in all subunits of all traces. Each edge is assigned a score or ‘weight’ to reflect how its pair of residues fits the electron density and how the pair would affect the quality of the final model. The calculation of edge weights is detailed in the next section. Once edge weights have been determined, the Bellman–Ford algorithm (Heineman *et al.*, 2008 ▶) is executed from the N-terminus to the C-terminus and from the C-­terminus to the N-terminus to find a path through the graph that minimizes the total weight. The trace that corresponds to the path of minimum weight is output. *Consensus­Modeler* was implemented in C++ and uses the Clipper libraries (Cowtan, 2000 ▶) for crystallographic computations.

#### 2.2.1. Edge weights   

Edge weights are heuristically derived values designed to identify and reward favorable features in a trace and penalize unfavorable features or errors such as mistracings and ‘frame-shifts’. The following features are taken into account.

(i) Agreement with the electron density. For each residue, agreement with the electron density is measured with a density cross-correlation coefficient computed by the algorithm.
(ii) Agreement with other residues. If other input traces have the same residue modeled at a spatial position, it is more likely to be correct than in cases where other traces have a different residue modeled. The number of similar residues at a single spatial position inversely contributes to the edge weight.
(iii) Geometry penalties. A penalty is incurred for dis­allowed Ramachandran values or whenever the distance of subsequent C^α^ atoms substantially deviates from the mean inter-peptide value.
(iv) Overlap penalty. Incorrectly traced backbones by model-building programs may result in multiple residues with distinct sequence numbers occupying the same spatial location after superposition. While the edge weight described under (ii) above increases the likelihood that the correct residue is inserted at the correct sequence position in the consensus model, it does not prevent an incorrectly traced residue at this spatial location also being included at its sequence position. This penalty aims to make overlapping residues mutually exclusive.
(v) Gaps. Analogous to sequence-alignment algorithms (*e.g.* Smith & Waterman, 1981 ▶), an output model can have gaps, *i.e.* missing fragments. Such a situation could arise if none of the input models have a residue modeled for the sequence position (often at the termini) or if any residue from the input models would result in an output model with higher score than a gap would. Gaps are modeled with ‘dummy’ residues. There is a high one-time gap-opening penalty and furthermore a lower penalty for each dummy residue to continue a gap.

#### 2.2.2. *Xpleo*    

The fragment-fitting software program *Xpleo* (available from http://smb.slac.stanford.edu/~vdbedem; van den Bedem *et al.*, 2005 ▶) was integrated with *ConsensusModeler*. Gaps of fewer than 15 residues in length in the consensus model are automatically identified, built and included.

#### 2.2.3. *qFit*    

The JCSG has recently developed an integer quadratic programming-based algorithm *qFit* (van den Bedem *et al.*, 2009 ▶) to identify and model alternate side-chain and main-chain conformations together with their occupancies. The software reduces subjectivity in assigning alternate con­formations and the resulting models show an improvement in the *R*_free_ statistic (Brünger, 1992 ▶). Work is under way to integrate *qFit* into *Xsolve*.

## 3. Results   

*Xsolve* was integrated into the JCSG structure-determination and refinement workflow. As part of the routine interaction with *Xsolve*, at termination the best traces are visually inspected by one of the JCSG’s staff crystallographers. Among these traces, one is selected to be uploaded to the JCSG’s tracking database to aid in further refinement. Generally, the longest trace with correctly docked side chains, *i.e.* the most complete trace, is selected.

By early 2010, nearly 770 structures had been deposited in the PDB. In 70% of cases an initial trace obtained from *ARP*/*wARP* was selected at a mean resolution of 1.8 Å. The remaining 30% were traced by *RESOLVE* 1 2 at a mean resolution of 2.1 Å. For the high-resolution quartile of data sets solved to better than 1.7 Å, *ARP*/*wARP* contributed 83% of the best traces, with *RESOLVE* contributing the remaining 17%. For the low-resolution quartile, *i.e.* data sets solved to worse than 2.1 Å, these proportions were 69 and 31%, respectively. The mean solvent contents were nearly identical at 49.8 and 50.7%, respectively. As expected, owing to the difference in mean resolution, the mean *R*_free_ for the *ARP*/*wARP* traces was slightly lower, at 0.205 *versus* 0.227.

Descriptive statistics were also computed for phasing software programs. It was found that 72% of the uploaded traces were phased with *SHARP* at a mean resolution of 1.88 Å and the remaining 28% were phased with* SOLVE* (mean resolution 1.91 Å). One out of three *RESOLVE* traces and one out of four *ARP*/*wARP* traces were phased with *SOLVE*. The slightly higher fraction for *RESOLVE* is possibly explained by these programs originating from the same author. The subsets of *SOLVE*- and *SHARP*-phased traces within the *RESOLVE* and *ARP*/*wARP* trace sets had identical mean resolutions, *i.e.* resolution outweighs the choice of phasing program for the efficacy of model-building programs.

The parallel exploration of parameters and processing strategies by *Xsolve* was particularly advantageous in solving challenging data sets. For instance, the JCSG solved 16 structures from twinned data sets using the MAD/SAD method, with twin fractions ranging from 0.14 to 0.48 (PDB entries 2i5i, 2p4g, 2pfw, 2pfx, 2prx, 2pyq, 2q02, 2q22, 3db2, 3duk, 3ejn, 3fxa, 3kst, 3mc3, 3b9t and 3lws). In these cases, traces were obtained from a solution in the apparent higher order space group as well as the correct space group and different phasing/density-modification/tracing program com­binations. In many instances structures were solved in both space groups, but often initial traces were better in the higher space group. Similarly, *Xsolve* has allowed the JCSG to evaluate multiple solutions when the initial space group is ambiguous.

### 3.1. Insights from reprocessing 36 data sets   

36 previously solved data sets were selected across various resolutions, space groups and sizes (Table 1 ▶).

**Figure 3:**
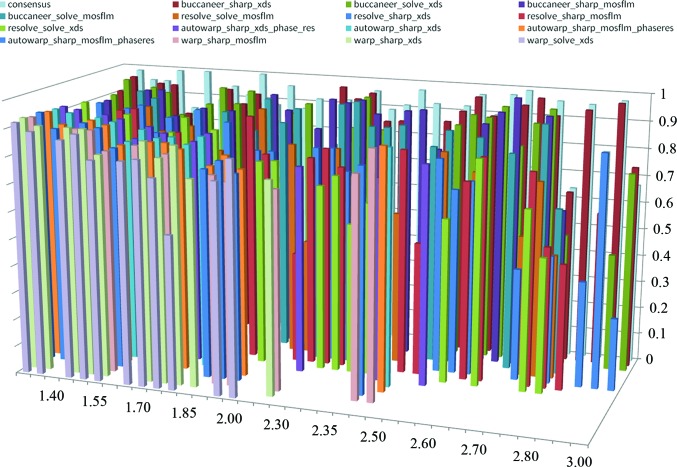
Fraction of residues from the sequence docked into the electron density for traces resulting from 36 reprocessed data sets. Depicted on the horizontal axis in the plane of the figure are the data sets, ranging in resolution from 1.3 to 3.0 Å. On the perpendicular horizontal axis are the processing strategies, with *ARP*/*wARP* traces in the foreground, *RESOLVE* traces in the middle and *Buccaneer* traces towards the back. The vertical axis represents the fraction of residues from the sequence docked into the electron density. Results are shown for the correct space group and number of molecules in the asymmetric unit and the most complete wavelength combination.

Fig. 3 ▶ graphically represents the completeness of traces resulting from distinct data-processing strategies in *Xsolve*. While the numbers of output traces are only shown for the correct content of the asymmetric unit and are summarized over wavelength combinations, the figure bears out that *Xsolve*’s volume of output exceeds what can efficiently be visually inspected in a high-throughput production environment. In 33 out of 36 cases (92%) *Buccaneer* reported the most complete trace. *ARP*/*wARP* traces, shown in the foreground, exhibit a high degree of completeness at higher resolutions. For the seven data sets at a resolution of 1.6 Å or better, two of the most complete traces resulted from *ARP*/*wARP*, one from *RESOLVE* and the remainder from *Buccaneer*. At lower resolution, *RESOLVE* (mid-section) and *Buccaneer* (background) provide traces that are more complete than those of *ARP*/*wARP* (Table 2 ▶).

**Figure 4:**
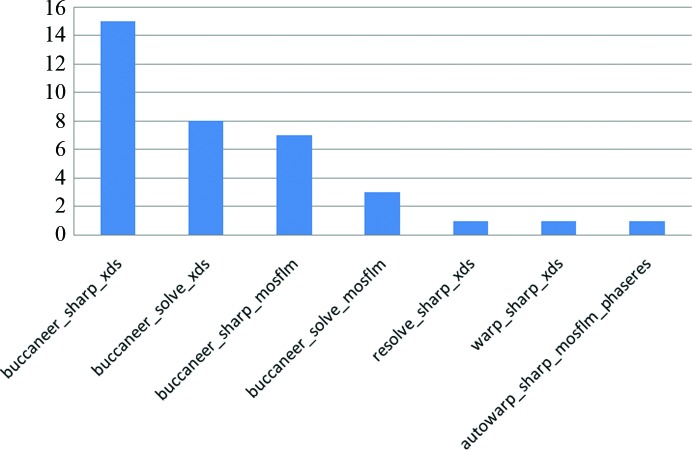
Number of times an indexing, phasing and model-building combination contributed the top trace for the 36 targets.

Particularly successful was the combination *XDS*/*SHARP*/*Buccaneer*, which accounted for 42% of all top traces (the greatest proportion of residues docked reported) across all resolutions (Fig. 4 ▶). However, all indexing, phasing and model-building software programs are represented among the top traces. Qualitatively, it was observed that while *Buccaneer* reports more complete traces, its error rate, *i.e.* fragments incorrectly docked into the density, tends to be higher than *RESOLVE*’s at lower resolution. *Buccaneer* is also fast, in one case tracing more than 3000 residues to 92% completeness in 90 min (PDB entry 3bjq; Table 1 ▶).

In *Xsolve*’s design, more important than a strategy’s ability to produce a top trace is simply that it differs from others and thus contains additional information. Manually evaluating each trace would be prohibitively labor-intensive, as *Xsolve* can produce dozens. *ConsensusModeler* capitalizes on the divergence in accuracy and completeness of input traces and computes a trace that is better than any input.

**Figure 5:**
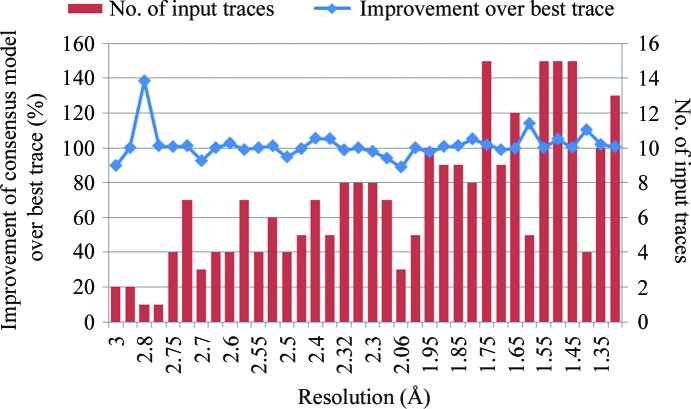
Percentage of improvement of the number of side chains docked to the electron density by the consensus model over the best input trace (blue line, left axis). The bars depict the number of input traces to *ConsensusModeler*. Only input traces with the correct space group and number of molecules in the asymmetric unit were considered. Wavelength combinations were binned, so that one input trace is reported for each program combination, similar to Fig. 3 ▶.

**Figure 6:**
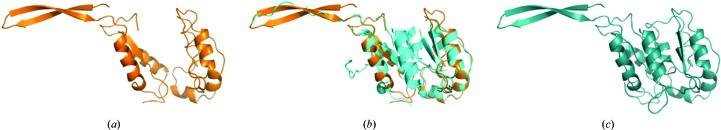
*ConsensusModeler* with two input models at 2.8 Å resolution. (*a*) The *Buccaneer*/*SHARP*/*XDS* model had 57% of the sequence docked into the model. (*b*) The *RESOLVE/SHARP*/*XDS* model had 39% of the sequence docked. (*c*) The consensus model resulted in 78% of the sequence docked.

Overall, *ConsensusModeler* provided a modest average improvement to the longest reported trace of about 1% additional residues docked into the electron density. Improvements in completeness are balanced by error correction of more aggressive traces. One in six traces improved more than 5% (Fig. 5 ▶), with a maximum of 38%. Indeed, occasionally traces that report lower completeness con­tain highly complementary parts. The crystal structure of a putative serine hydrolase (NP_639225.1) from *Xanthomonas campestris* was solved at 2.8 Å resolution (PDB entry 3ksr). In this case, *Xsolve* output only two traces: a *Buccaneer* trace with 57% of the residues docked and a *RESOLVE* trace with 39% of the residues docked. These highly complementary traces were com­bined by *ConsensusModeler* into a trace that was 78% complete (Fig. 6 ▶).

Five of the 36 consensus models had fewer than 95% of the residues docked compared with the best trace (Fig. 5 ▶). Of these five, two have not resulted in a PDB deposition as none of the *Xsolve* traces or consensus models were deemed sufficiently complete and accurate to proceed with refinement (YP_001197814.1 and NP_388303.1). Closer examination of the other three revealed that in one case *ConsensusModeler* had successfully omitted two incorrectly traced long fragments (in 3mcp), while in others it had erroneously removed a helix (in 3k9i) and a 20-residue fragment (in 3dkq).

**Figure 7:**
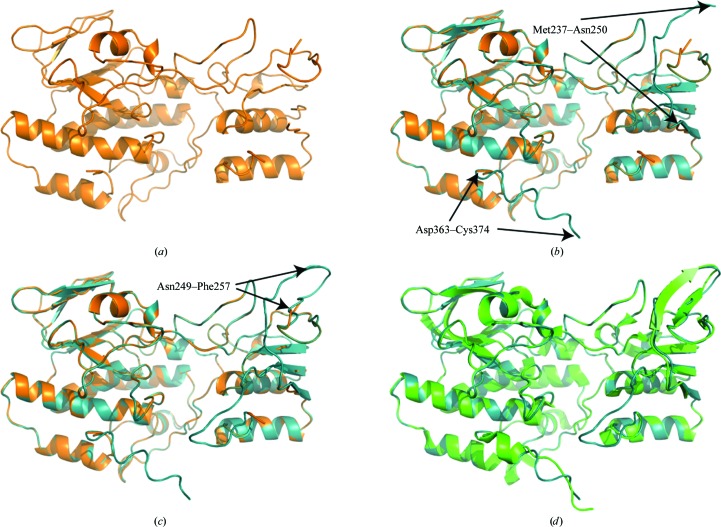
*ConsensusModeler*-facilitated *Xpleo*. (*a*) The *Buccaneer* model had 92% of side chains docked. (*b*) The consensus model (cyan) calculated from seven input traces, with an 11-residue fragment added to the C-terminus and partially closing a 20-residue fragment from Met237 to Phe257. (*c*) *Xpleo* was able to fully close the remaining eight-residue gap from Asn249 to Phe257, resulting in a trace with 97% of residues docked. (*d*) The final refined model in green superimposed on the consensus model.

*ConsensusModeler* also facilitated *Xpleo* by partially closing gaps in input traces. The remaining missing fragments were easily computed and fitted to the electron density with *Xpleo*. The structure of a putative oxygenase (YP_001051978.1) from *Shewanella baltica* OS155 was solved at 2.3 Å resolution (PDB entry 3dkq). *Buccaneer* reported the most complete trace, with 92% of side chains docked (Fig. 7 ▶ *a*). A consensus model was calculated from seven input traces, with the least complete model having 56% of side chains docked. The consensus model added an 11-residue fragment to the C-terminus and a 13-residue fragment from Met237 to Asn250 and thus partially closed a 20-residue gap (Fig. 7 ▶ *b*, cyan). *Xpleo* was able to fully close the remaining eight-residue gap from Asn249 to Phe257, resulting in a trace with 97% of residues docked (Fig. 7 ▶ *c*). Fig. 7 ▶(*d*) shows the final refined model in green superimposed on the consensus model.

## 4. Conclusions   

*Xsolve*’s design to semi-exhaustively and in parallel explore key parameters of the structure-determination process and to utilize multiple software programs increased efficiency and resulted in high-quality traces. For the 36 data sets that we examined in detail, all indexing, phasing and model-building programs resulted in a top trace and furthermore always contributed to a consensus model. This validates *Xsolve*’s approach to run all parameter and software combinations to termination rather than choosing an optimal strategy. A consensus model can provide an optimal starting point for subsequent manual refinement. *ConsensusModeler* is most effective when input models exhibit variation in completeness and accuracy. Aggressive model-building efforts by some programs, resulting in higher model completeness at the expense of elevated tracing errors, can be offset by a more conservative approach employed by others.

*Xsolve* has been instrumental to structure determination at scale, allowing the JCSG to deposit 200 high-quality structures per year in the PDB for the last few years. While parallelism inevitably results in some computational overhead, *Xsolve*’s run-time on each single data set generally does not exceed that of the slowest combination of programs. The model-building stage is the slowest step, ranging from a few hours to several days for large structures. Once completed, the median number of calendar days to refine an initial set of coordinates from *Xsolve* was only seven. Furthermore, the JCSG’s structures scored highly in an independent broad quality survey (Brown & Ramaswamy, 2007 ▶).

It should be emphasized that all software programs in *Xsolve* are run with default parameter settings. The results reported in §[Sec sec3]3 for model-building and phasing programs are therefore not representative of those that could be obtained by a skilled crystallographer using the same programs.

## Supplementary Material

Supporting information file. DOI: 10.1107/S0907444910039934/ba5156sup1.pdf

## Figures and Tables

**Table 1:** Characteristics of the 36 data sets selected for reprocessing The information in this table may differ from that displayed in the PDB archive. For some targets data were collected from an additional crystal to facilitate refinement.

PDB code	Space group	Sequence length	Molecules in asymmetric unit	Resolution ()
3mcp	*P*4_3_2_1_2	365	1	3.0
3n0v	*P*2_1_	285	4	2.89
3ksr	*P*4_3_2_1_2	289	1	2.8
3ec4	*P*2_1_2_1_2_1_	227	2	2.8
3k8r	*I*4	123	2	2.75
3cuc	*P*3_1_21	290	2	2.71
3k9i	*P*6_1_22	117	1	2.71
3do5	*C*222_1_	326	1	2.7
3bjq	*P*2_1_	315	10	2.6
2qdr	*P*6_2_22	302	2	2.6
3dxq	*P*2_1_2_1_2	300	2	2.55
2re3	*P*4_3_2_1_2	193	2	2.53
YP_001197814.1	*P*6_1_22	236	1	2.5
3knz	*P*2_1_2_1_2_1_	347	6	2.5
3d1c	*P*4_3_2_1_2	368	1	2.4
2op5	*P*2_1_2_1_2_1_	116	6	2.35
3dxp	*P*2_1_2_1_2	358	1	2.32
3dde	*P*2_1_22_1_	238	2	2.3
3d7q	*P*4_3_2_1_2	111	2	2.3
3dkq	*P*4_1_2_1_2	224	3	2.26
NP_388303.1	*P*2_1_2_1_2_1_	140	1	2.06
3k6o	*C*2	223	2	2
3htv	*C*222_1_	309	1	1.95
3gyc	*P*2_1_	392	2	1.85
3kog	*I*2_1_2_1_2_1_	255	1	1.85
3fcr	*C*2	457	1	1.8
2p7i	*I*422	249	2	1.75
2pfx	*P*6_3_	190	2	1.7
2qeu	*P*6_1_22	140	3	1.65
2ou5	*P*2_1_2_1_2_1_	174	2	1.6
3f8x	*P*1	147	4	1.55
3hdx	*P*4_1_2_1_2	477	1	1.5
3gr3	*P*2_1_2_1_2_1_	229	2	1.45
3isx	*C*2	331	1	1.4
2qjw	*P*2_1_	175	4	1.35
3hwu	*H*3	146	1	1.3

**Table 2:** Average percentage of side chains docked into electron density for each of the three model-building programs and the number of data sets for which it produced the most complete trace The average was calculated over all models that had 40% or more of side chains docked.

	*ARP*/*wARP*	*RESOLVE*	*Buccaneer*
All resolutions (%)	84.7	72.7	89.5
Better than 1.8 (%)	87.1	83.3	89.2
Worse than 2.5 (%)	0	60.1	87.2
No. of top traces	2	1	33

## Footnotes

1 Supplementary material has been deposited in the IUCr electronic archive (Reference: BA5156). Services for accessing this material are described at the back of the journal.

2
*Buccaneer* was added to *Xsolve* early in 2010 and has been excluded from the analysis.
