# Supplementary material for: Distributed structure determination at the JCSG
Source: Acta Crystallogr D Biol Crystallogr. 2011 Mar 18;67(Pt 4):368–75. doi: 10.1107/S0907444910039934 (PMC3069752; doi:10.1107/S0907444910039934)
Supplement: Supplementary file 1 [file d-67-00368-sup1.pdf]

JCSG Data Collection

http://sdc08.slac.stanford.edu:3000/collection/edit/16297

Suggested Sites | Web Slice Gallery | Structure refinemen... | Import to Mendeley | Other bookmarks

# Editting Data

HP10645F 137473_2 Show | Collection List | Delete | Xsolve | Xsolve Upload | Processing Results

| | |
|---|---|
| **Date Data Collected:** | 2009-12-04 |
| **Beamline:** | BL11_1 |
| **Target:** | HP10645F |
| **Crystal ID:** | 137473_2 |
| **Experiment:** | SAD |
| **Resolution:** | 2.1 |
| **Data Collected by:** | Jessica |
| **Image Directory:** | /data/jcsg/ssrl4/11_1/20091202/collection/HP10645F/137473 |
| **HKL2000 Spotsize:** | |
| **Autoindex Images:** | Prefix: Image1: Image2: |
| **Beam Center:** | BeamX: BeamY: |
| **Lambda1:** | fp: -7.2695 fpp: 5.2442 prefix list: 137473_1 |
| **Lambda2:** | fp: fpp: prefix list: |
| **Lambda3:** | fp: fpp: prefix list: |
| **Lambda4:** | fp: fpp: prefix list: |
| **Space Group List:** | P1 |
| **Number of Molecules:** | |
| **Comments:** | 04 Dec 2009 10:59:22 AM stog_operation_completed madScan 46.175 normal 12657.330078 -9.360322 3.041869 12663.870117 -7.269518 5.244236 13500.000000 -1.800000 3.400000 NULL/137473scan 137473smooth_exp.bip 137473smooth_norm.bip 137473fp_fpp.bip<br>04 Dec 2009 10:59:22 AM Inflection Energy: 12657.330078 eV<br>04 Dec 2009 10:59:22 AM Inflection f' : -9.360322<br>04 Dec 2009 10:59:22 AM Inflection f'' : 3.041869 |

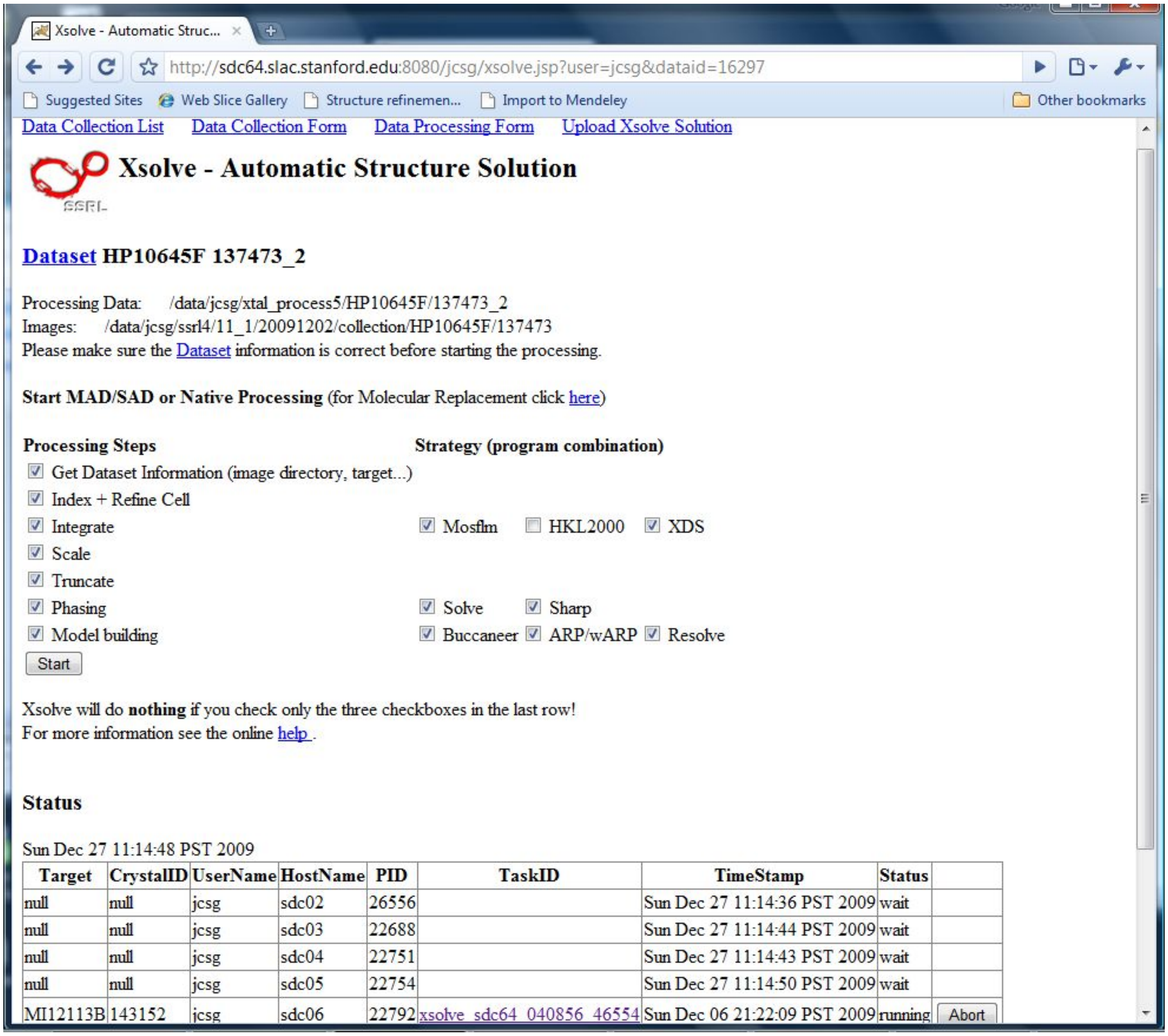

Xsolve - Automatic Struc...

http://sdc64.slac.stanford.edu:8080/jcsg/xsolve.jsp?user=jcsg&dataid=16297

Suggested Sites | Web Slice Gallery | Structure refinemen... | Import to Mendeley | Other bookmarks

Data Collection List   Data Collection Form   Data Processing Form   Upload Xsolve Solution

# Xsolve - Automatic Structure Solution

SSRL

## Dataset HP10645F 137473_2

Processing Data: /data/jcsg/xtal_process5/HP10645F/137473_2
Images: /data/jcsg/ssrl4/11_1/20091202/collection/HP10645F/137473
Please make sure the Dataset information is correct before starting the processing.

**Start MAD/SAD or Native Processing** (for Molecular Replacement click here)

| Processing Steps | Strategy (program combination) |
|---|---|
| ☑ Get Dataset Information (image directory, target...) | |
| ☑ Index + Refine Cell | |
| ☑ Integrate | ☑ Mosflm ☐ HKL2000 ☑ XDS |
| ☑ Scale | |
| ☑ Truncate | |
| ☑ Phasing | ☑ Solve ☑ Sharp |
| ☑ Model building | ☑ Buccaneer ☑ ARP/wARP ☑ Resolve |

Start

Xsolve will do **nothing** if you check only the three checkboxes in the last row!
For more information see the online help .

## Status

Sun Dec 27 11:14:48 PST 2009

| Target | CrystalID | UserName | HostName | PID | TaskID | TimeStamp | Status | |
|---|---|---|---|---|---|---|---|---|
| null | null | jcsg | sdc02 | 26556 | | Sun Dec 27 11:14:36 PST 2009 | wait | |
| null | null | jcsg | sdc03 | 22688 | | Sun Dec 27 11:14:44 PST 2009 | wait | |
| null | null | jcsg | sdc04 | 22751 | | Sun Dec 27 11:14:43 PST 2009 | wait | |
| null | null | jcsg | sdc05 | 22754 | | Sun Dec 27 11:14:50 PST 2009 | wait | |
| MI12113B | 143152 | jcsg | sdc06 | 22792 | xsolve_sdc64_040856_46554 | Sun Dec 06 21:22:09 PST 2009 | running | Abort |

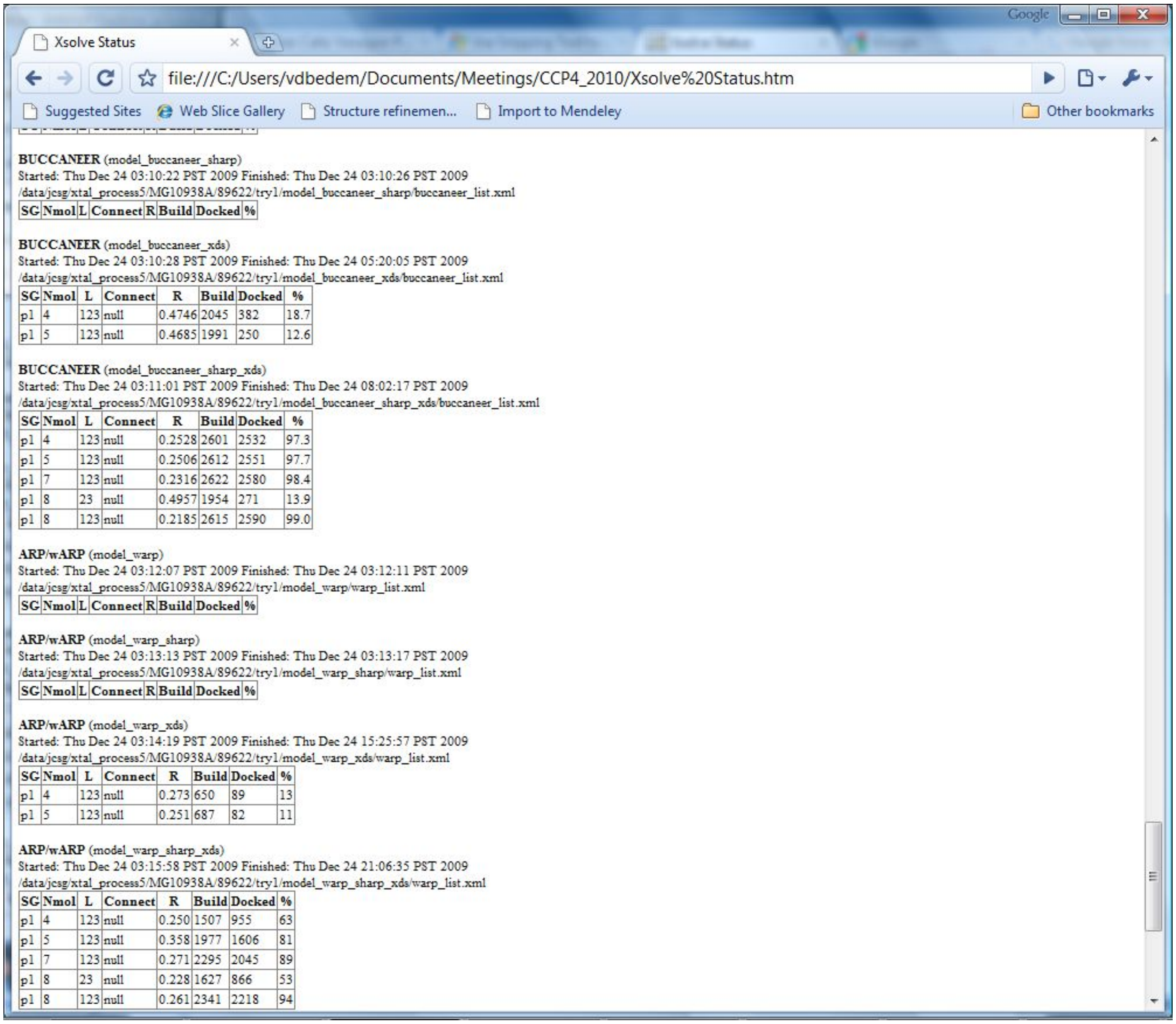


**User Interface for Xsolve. The web page at the top gathers information about the experiment. Xsolve is started from a web interface as well, shown in the middle. At the bottom an example of a status page is shown.**
